# A comparison study of dynamic [^18^F]Alfatide II imaging and [^11^C]MET in orthotopic rat models of glioblastoma

**DOI:** 10.1007/s00432-024-05688-4

**Published:** 2024-04-22

**Authors:** Yue Pan, Haodan Dang, Haoxi Zhou, Huaping Fu, Shina Wu, Huanhuan Liu, Jinming Zhang, Ruimin Wang, Yuan Tian, Baixuan Xu

**Affiliations:** 1 Chinese PLA General Hospital, Chinese PLA Medical School, Beijing, China; 2 Department of Nuclear Medicine, First Medical Center of Chinese PLA General Hospital, Fuxing Road 28, Beijing, 100853 China; 3 Department of Radiology, The 8th Medical Center of Chinese PLA General Hospital, Beijing, China

**Keywords:** Glioblastoma multiforme (GBM), [^18^F]Alfatide II, [^11^C]Methionine([^11^C]MET), Positron emission tomography (PET)

## Abstract

**Purpose:**

To investigate and compare the dynamic positron emission tomography (PET) imaging with [^18^F]Alfatide II Imaging and [^11^C]Methionine ([^11^C]MET) in orthotopic rat models of glioblastoma multiforme (GBM), and to assess the utility of [^18^F]Alfatide II in detecting and evaluating neoangiogenesis in GBM.

**Methods:**

[^18^F]Alfatide II and [^11^C]MET were injected into the orthotopic GBM rat models (*n* = 20, C6 glioma cells), followed by dynamic PET/MR scans 21 days after surgery of tumor implantation. On the PET image with both radiotracers, the MRI-based volume-of-interest (VOI) was manually delineated encompassing glioblastoma. Time-activity curves were expressed as tumor-to-normal brain ratio (TNR) parameters and PET pharmacokinetic modeling (PKM) performed using 2-tissue-compartment models (2TCM). Immunofluorescent staining (IFS), western blotting and blocking experiment of tumor tissue were performed for the validation.

**Results:**

Compared to ^11^C-MET, [^18^F]Alfatide II presented a persistent accumulation in the tumor, albeit with a slightly lower SUVmean of 0.79 ± 0.25, and a reduced uptake in the contralateral normal brain tissue, respectively. This resulted in a markedly higher tumor-to-normal brain ratio (TNR) of 18.22 ± 1.91. The time–activity curve (TACs) showed a significant increase in radioactive uptake in tumor tissue, followed by a plateau phase up to 60 min for [^18^F]Alfatide II (time to peak:255 s) and 40 min for [^11^C]MET (time to peak:135 s) post injection. PKM confirmed significantly higher K_1_ (0.23/0.07) and K_3_ (0.26/0.09) in the tumor region compared to the normal brain with [^18^F]Alfatide II. Compared to [^11^C]MET imaging, PKM confirmed both significantly higher K_1_/K_2_ (1.24 ± 0.79/1.05 ± 0.39) and K_3_/K_4_ (11.93 ± 4.28/3.89 ± 1.29) in the tumor region with [^18^F]Alfatide II. IFS confirmed significant expression of integrin and tumor vascularization in tumor region.

**Conclusion:**

[^18^F]Alfatide II demonstrates potential in imaging tumor-associated neovascularization in the context of glioblastoma multiforme (GBM), suggesting its utility as a tool for further exploration in neovascular characterization.

**Supplementary Information:**

The online version contains supplementary material available at 10.1007/s00432-024-05688-4.

## Introduction

Glioblastoma multiforme (GBM), the most malignant form of astrocytoma, represents a paramount challenge in neuro-oncology due to its aggressive spreading growth pattern and complex pathophysiology (Burko et al. 2023; Wang et al. 2023). The current standard treatment modalities for GBM include surgical resection followed by adjuvant radiation therapy and chemotherapy with temozolomide (Clavreul et al. 2022; Raue et al. 2023). Despite these interventions, the clinical outcome for GBM patients remains poor (Petkovic et al. 2023). It ranks as a leading cause of mortality associated with brain tumors in the adult population, characterized by a median survival duration of approximately 12 months post-diagnosis and a five-year survival rate lingering around 5%, but the recurrence rate is close to 100% (Schaff and Mellinghoff 2023).

Recent advancements in imaging techniques have significantly enhanced our understanding and management of GBM. Magnetic resonance (MR) imaging, especially with advanced modalities like functional MRI sequences include and diffusion kurtosis imaging (DKI) and perfusion weighted imaging (PWI), has provided deeper insights into the tumor’s anatomy and physiology (Zhuang et al. 2022). Positron emission tomography (PET) imaging, coupled with novel radiotracers, has also made significant strides in GBM research. Tracers like [^11^C]Methionine ([^11^C]MET), an amino acid PET tracer, have shown promise in better identifying GBM extent and heterogeneity, as well as in monitoring treatment response (Galldiks et al. 2022; Nakajo et al. 2022). Despite advances in [^11^C]MET PET imaging, it is important to acknowledge its inherent limitations. The relatively short half-life of [^11^C], approximately 20 min, which limits the widespread availability and practical use of [^11^C]MET (Michaud et al. 2020). Additionally, the increased uptake of [^11^C]MET can also be observed in areas of inflammation, infection, and even in some benign brain lesions, which may lead to false positive diagnosis of GBM (Kim et al. 2022). This is also a challenge in accurately assessing treatment response and identifying tumor recurrence, particularly in radiation encephalopathy or pseudoprogression (Dang et al. 2022; Pessina et al. 2021; van Dijken et al. 2022).

On the other hand, [^18^F]Alfatide II, a radiolabeled peptide based on the molecular structure of arginine–glycine–aspartic acid (RGD), which is a key component for targeting integrins. Integrins, specifically the αvβ3 subtype, are cell surface receptors that play a crucial role in cell adhesion, migration, and angiogenesis. The expression of integrin αvβ3 is significantly upregulated in various tumor types, including glioblastoma (GBM), and is particularly associated with tumor angiogenesis and metastatic potential (Bao et al. 2016). Although there has been research on [^18^F]Alfatide II in the context of glioblastoma multiforme (GBM), studies focusing on dynamic PET semi-quantitative analysis and the kinetic parameters of [^18^F]Alfatide II remain limited (Zhang et al. 2016). Additionally, comparative studies diagnosing GBM with [^18^F]Alfatide II and [^11^C]MET are scarce.

In our study, we aimed to explore the comprehensive comparison of dynamic PET imaging with [^18^F]Alfatide II and [^11^C]MET in orthotopic rat models of GBM, and to assess the appropriateness of [^18^F]Alfatide II PET imaging for GBM.

## Materials and methods

### Animals

All animal experiments were approved by the ethics committee and institutional review board of our hospital. All SPF grade male Wistar rats from Charles River Laboratories (Beijing, China) aged 6–8 weeks were fed at the temperature of 18–25 ℃, with relative humidity of 35–70 ℃ and a 12-h day-to-day cycle to provide the rats with adequate food and drinking water and environmental enrichment. The animals were allowed a 7-day acclimatization period before the experiments.

### Orthotopic animal model of GBM

C6 glioma cells from GuYan Biotech Co., Ltd. (Shanghai, China) were cultured in DMEM at 37 °C and 5% CO2. After two generations of cell culture, C6 cells were trypsinized and resuspended at the concentration of 1 × 10^7^ cells/mL to prepare GBM model. Rats (*n* = 23) were anesthetized with 2% pentobarbital sodium (0.2 ml/100 g), and then their heads were fixed on a brain stereotactic instrument. After the hair on the top of the head was cut off, a small hole with a diameter of about 2 mm was drilled at the intersection of the left side of sagittal suture 3 mm and the front side of coronal suture 1 mm. Next, 10 μL of C6 cell suspension with a concentration of 1 × 10^7^ cells /mL was injected into the brain with a 0.25 mL microinjector at a speed of 1 μL/min. After administering the injection, the syringe was carefully withdrawn. The burr hole was then sealed with bone wax, followed by suturing the skin closed.

### Preparation of radiotracers

The prodrug NOTA-PEG4-E [c (RGDfK)2] kit was purchased from Ruida Fuming Technology Co., Ltd. The ^18^F-fluoride in O-18 water was obtained from a cyclotron (HM-20 cyclotron, Sumitomo Heavy Machinery Co., Ltd., China People’s Liberation Army General Hospital). The radiolabeling of NOTA-PEG4-E [c (RGDfK)2] and [^18^F]AlF was performed following the previously published procedure with some modifications (Guo et al. 2014b). The final product was designated [^18^F]Alfatide II ([^18^F]-ALF-NOTA-E [PEG 4-c (RGDFK)] 2). [^11^C]methionine was synthesized by online [^11^C]methylation of L-homocysteine on GE TRACERlab FX-C Pro module. The purity of [^18^F]Alfatide II and [^11^C]methionine was determined by HPLC.

### PET imaging of GBM rat model

The rats, following tumor cell implantation for 14 days, were anesthetized and scanned on a whole-body PET/MR scanner (SIGNA™ PET/MR, GE Healthcare, Chicago, USA). The animals were imaged with 3T MR imaging using a mouse brain radiofrequency coil by applying T1-weighted imaging and T2-weighted imaging (fast spin echo, FSE). Simultaneous dynamic PET and MRI was performed with 150 mm field of view for whole-body mouse imaging. For [^18^F]Alfatide II imaging, the rat models received the intravenous bolus injection of 13.0 ± 2.1 MBq of the radiotracer. This was followed by a comprehensive 60-min dynamic scan, executed in list mode. For the [^11^C]MET imaging procedure, a similar approach was adopted with the intravenous bolus injection of 15.0 ± 2.7 MBq and a 40-min dynamic scan. We conducted [^11^C]MET and [^18^F]Alfatide II PET/MR scans on 20 rats, with a time interval of no more than 48 h between the scans using the two tracers.

The list-mode data were reconstructed into 1 × 5 s, 1 × 25 s, 9 × 30 s, 5 × 60 s, 5 × 120 s and 10 × 240 s for [^18^F]Alfatide II and 1 × 5 s, 1 × 25 s, 9 × 30 s, 5 × 60 s, 5 × 120 s and 5 × 240 s for [^11^C]MET, respectively, using 3D ordered subset expectation maximization with 1 iteration, 32 subsets, and a VOXEL size of 0.42 mm, applying correction for random coincidences, decay, deadtime, and scatter correction.

### PET data analysis

On the PET image with both radiotracers, the MRI-based volume-of-interest (VOI) was manually delineated encompassing glioblastoma. A spherical VOI of constant size (diameter, 10 mm) was positioned in normal brain tissue, including grey and white matter, in the contralesional hemisphere, liver and muscle. The mean standardized uptake value (SUVmean) of the VOI was measured, based on static PET/MR images taken 60 min for [^18^F]Alfatide II or 40 min for [^11^C]MET after injection. Time-activity curve (TACs) was obtained by VOI in each time frame of the whole 60 min ([^18^F]Alfatide II) or 40 min ([^11^C]MET) dynamic image sequence. The following parameters were extracted from TACs: time to peak (TTP) and slope of curve.

### Kinetic modeling

The Two-tissue-compartment models (2TCM) was used to describe tracer kinetics of [^18^F]Alfatide II and [^11^C]MET, where C_p_ denotes the concentration of tracer in arterial plasma, C_t_ represents the interstitial and intracellular free or non-specific binding components, and C_m_ signifies the specific components of the tracer. The transport and binding rates of the tracer are described as follows: The transfer from arterial plasma to tissue (K_1_ [mL/g/min]), tissue clearance (K_2_ [1/min]), the on-rate of specific binding (K_3_ [1/min]), and the target dissociation rate (K_4_ [1/min]).

Model equations are illustrated as:

1 $$\frac{d{C}_{t}(t)}{d(t)}={K}_{1}{C}_{P}\left(t\right)-\left({K}_{2}+{K}_{3}\right){C}_{t}\left({\text{t}}\right)+{K}_{4}{C}_{m}\left({\text{t}}\right)$$

2 $$\frac{d{C}_{m}(t)}{d(t)}={K}_{3}{C}_{t}\left(t\right)-{K}_{4}{C}_{m}\left({\text{t}}\right)$$

The values of K_1_–K_4_ are determined by fitting the model to the time–activity curve. The model equation was calculated quantitatively using the left ventricular region of interest (ROI) as C_P_, representing the arterial blood input function. To optimize the fit, Akaike information criterion (AIC) was employed for evaluating its efficiency (Watabe et al. 2006).

In terms of pharmacokinetics, the volume of distribution (V_T_) can be categorized into two components: specific volume of distribution (V_S_) and non-displaceable volume (V_ND_), which represents the nonspecific distribution:

3 $${V}_{S}=\frac{{K}_{1}{K}_{3}}{{K}_{2}{K}_{4}}$$

4 $${V}_{ND}={\frac{{K}_{1}}{{K}_{2}}}$$

5 $${V}_{T}={V}_{S}+{V}_{ND}={\frac{{K}_{1}}{{K}_{2}} (1+\frac{{K}_{3}}{{K}_{4}}) }$$

All calculations were performed by the analysis software (PMOD v.4.3, PMOD technologies).

### Histological analysis

For validation of the PET image-based results immunofluorescent staining, western blotting and Biodistribution study were performed as described in the Supplemental Information. The blocking studies were carried out in 3 Orthotopic rat models of GBM. After injecting [^18^F]Alfatide II for 60 min, PET imaging was performed. Two days later, imaging was performed 60 min after co-injection of unlabeled RGD (2 mg/kg) and [^18^F]Alfatide II (13.0 ± 3.1 MBq).

### Statistical analysis

Numerical data are presented as mean ± standard deviation (SD). GraphPad (version 5.03, GraphPad Software, San Diego, Calif) was used for the paired two-sample t test and was performed to compare SUV values. *P* < 0.05 was considered to indicate a significant difference.

## Results

### Radiosynthesis of the radiotracers

For [^18^F]Alfatide II, the purity of the synthesized product is 99.9%. For [^11^C]MET, the purity of the synthesized product is 99.0%. (Supplement Fig. 1).

### Semi‑quantitative analysis of dynamic PET imaging

**Fig. 1:**
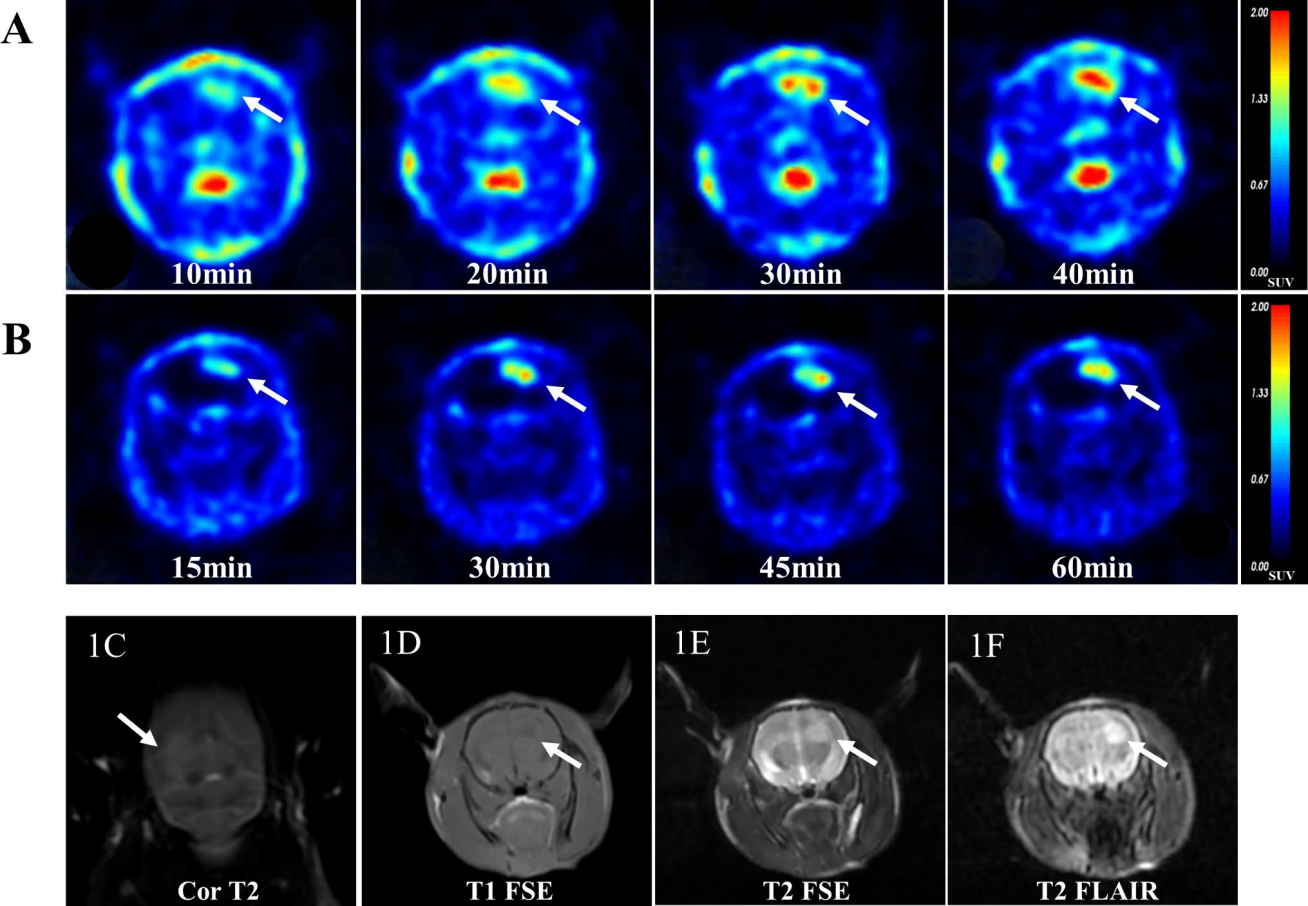
PET/MR imaging of the GBM rat model with ^11^C-MET and [^18^F]Alfatide II at various time points. **A** PET images of C6 tumor-bearing rat at 10, 20, 30, and 40 min after injection of 15 MBq of ^11^C-MET. Tumors are indicated by arrows. **B** PET images of C6 tumor-bearing rat at 15, 30, 45, and 60 min after injection of 13 MBq of [^18^F]Alfatide II. Tumors are indicated by arrows. The MR imaging shows that the tumor areas are clearly depicted as high signal intensity on Cor T2 (**C**), T2 FSE (**D**), and T2 FLAIR (**F**), while appearing as low signal intensity on T1 FSE (**E**)

**Fig. 2:**
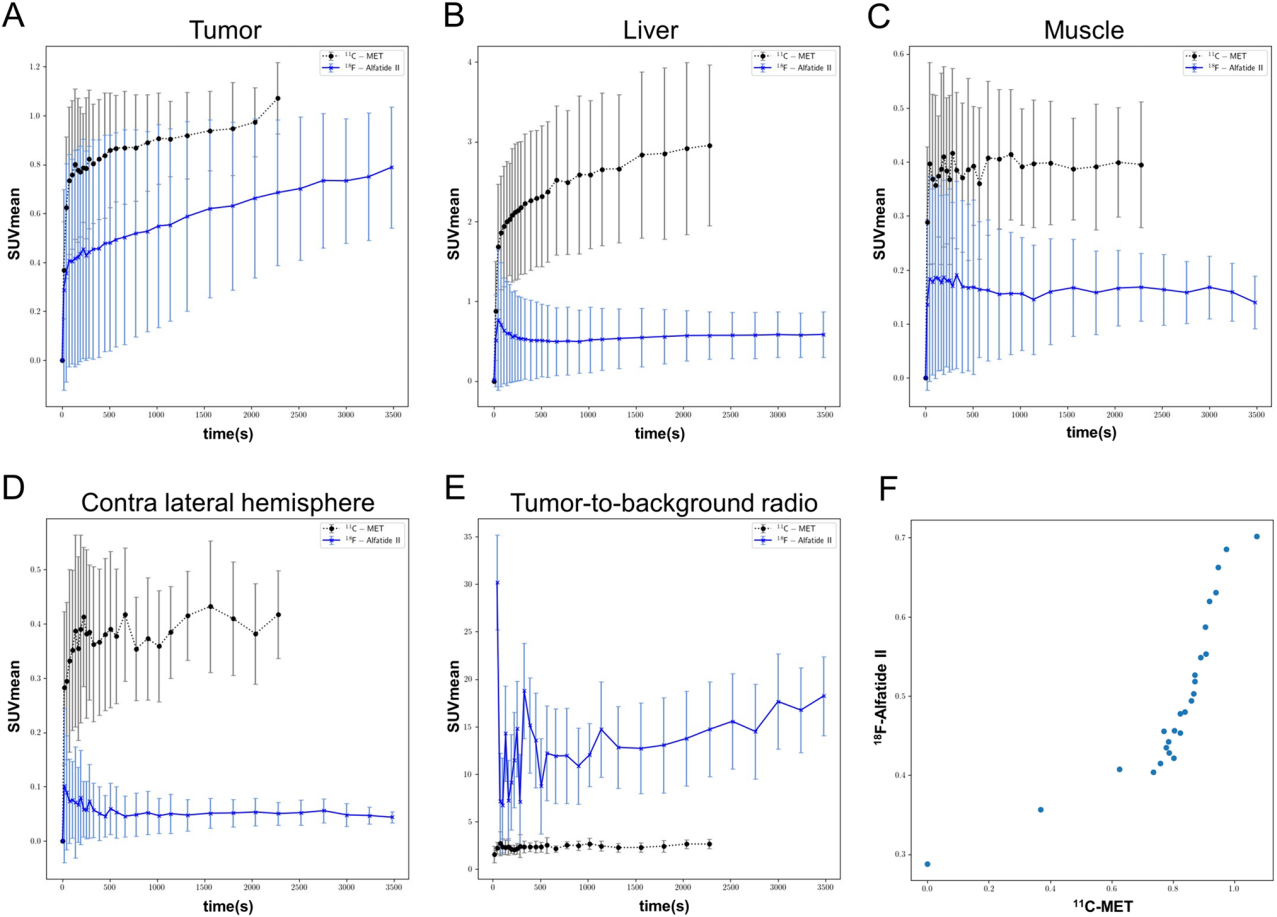
Dynamic uptake (SUVmean ± SD) following injection of ^11^C-MET and [^18^F]Alfatide II (*n* = 20) after 14 days of tumor growth in the tumor region (**A**), liver (**B**), muscle (**C**) and the contra lateral hemisphere (**D**). **E** TBR based on SUVmean using the contra lateral hemisphere as reference. **F** Scatter plot of Pearson’s correlation (*r*) comparing tumor uptake of ^11^C-MET and [^18^F]Alfatide II

Both the radiotracers of [^18^F]Alfatide II and [^11^C]MET demonstrated enhanced delineation of the tumor tissue visually (Fig. 1). The tumor demonstrated a notably higher uptake of [^11^C]MET, reaching an SUVmean of 1.07 ± 0.15(0.83, 1.30) at the 40 min. Similarly, [^18^F]Alfatide II showed a persistent accumulation in the tumor, albeit with a slightly lower SUVmean of 0.79 ± 0.25(0.45, 1.63) observed after 60 min (Fig. 2A). Crucially, compared to [^11^C]MET, [^18^F]Alfatide II presented a reduced uptake in the contralateral normal brain tissue, registering values of 0.39 ± 0.12(0.22, 0.71) (11C-MET) and 0.14 ± 0.05(0.07, 0.27) ([^18^F]Alfatide II), respectively. This resulted in a markedly higher tumor-to-normal brain ratio (TNR) of 6.0 ± 1.91(3.05, 11.12) for [^18^F]Alfatide II, compared to [^11^C]MET (2.91 ± 0.81)(1.62, 3.96), as detailed in Fig. 2. There was a high correlation (Pearson’s *r* = 0.799) between [^18^F]Alfatide II and [^11^C]MET uptake in the tumor region (Fig. 2F).The time–activity curve (TACs) showed a significant increase in radioactive uptake in tumor tissue, followed by a plateau phase up to 60 min for [^18^F]Alfatide II and 40 min for [^11^C]MET post injection. The TACs of the tumor-to-background revealed that the peak uptake of [^18^F]Alfatide II occurred at 255 s, whereas that of [^11^C]MET was observed at 135 s (Fig. 2E).

### Quantitative analysis of dynamic PET imaging

Table 1 presented all the rate constants of both radiotracers. For [^18^F]Alfatide II, in the tumor area, both K_1_ (0.23 ± 0.16) and K_3_ (0.26 ± 0.26) were significantly higher than in the normal brain region. Similar features were also shown in the [^11^C]MET imaging. Compared to [^11^C]MET imaging, PKM confirmed both significantly higher K_1_/K_2_ (1.24 ± 0.79/1.05 ± 0.39) and K_3_/K_4_ (11.93 ± 4.28/3.89 ± 1.29) in the tumor region with [^18^F]Alfatide II.

**Table 1:** Kinetic parameters of [^18^F]Alfatide II and [^11^C]MET

		K_1_	K_2_	K_3_	K_4_	K_1_/K_2_	K_3_/K_4_	V_S_	V_T_
[^18^F]Alfatide II	GBM	0.23 ± 0.16	0.43 ± 0.21	0.26 ± 0.26	0.33 ± 0.30	1.24 ± 0.79	11.93 ± 4.28	6.16 ± 1.80	7.40 ± 2.54
	Normal brain tissue	0.07 ± 0.02	0.07 ± 0.02	0.09 ± 0.07	0.60 ± 0.33	0.24 ± 0.06	7.73 ± 1.98	1.55 ± 0.15	1.78 ± 0.16
[^11^C]MET	GBM	1.78 ± 0.16	0.54 ± 0.14	0.24 ± 0.06	0.40 ± 0.25	1.05 ± 0.39	3.89 ± 1.29	2.68 ± 0.44	3.74 ± 0.47
	Normal brain tissue	0.16 ± 0.06	0.23 ± 0.09	0.06 ± 0.02	0.14 ± 0.06	0.76 ± 0.30	2.80 ± 1.12	2.27 ± 0.91	3.03 ± 1.21

### Validation of results

**Fig. 3:**
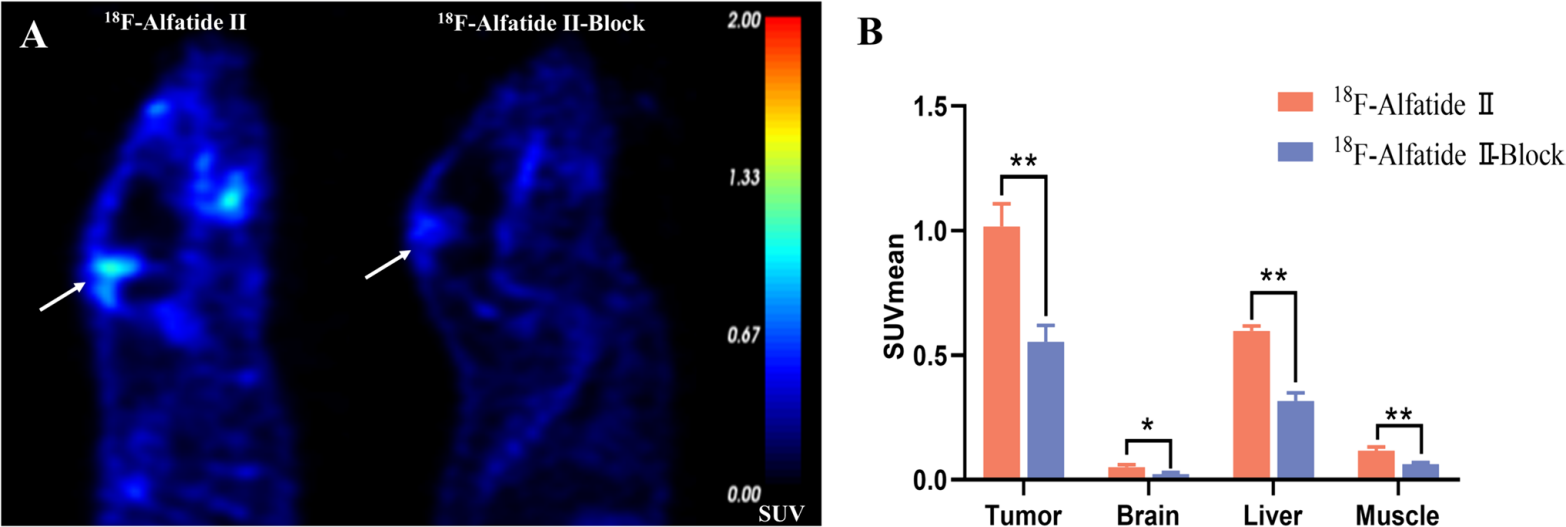
Results of blocking in three rat models of glioblastoma: **A** The PET images of non-blocking group and blocking group 60 min after injection of [^18^F]Alfatide II. **B** The tumor and other tissues uptake of [^18^F]Alfatide II in the blocking group was significantly reduced, compared with the non-blocking group. The SUVmean of the tumor was reduced from 1.02 ± 0.09 to 0.55 ± 0.07. The two tumors are indicated with white arrows and the color scale is expressed in SUV values. *p < 0.05 for paired comparisons

**Fig. 4:**
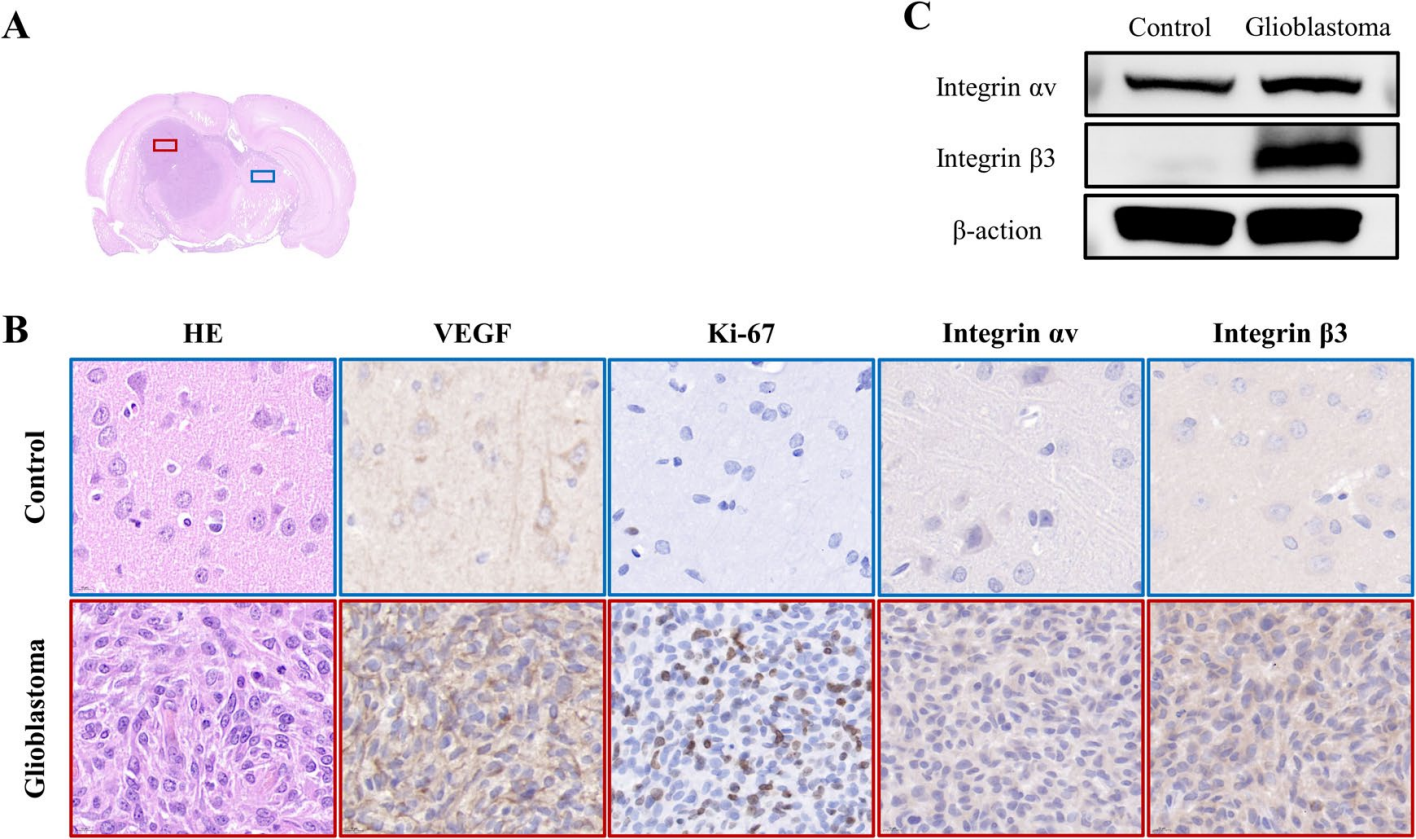
**A** Diagram of glioblastoma brain section showing the location of tumor (red area) and normal control region (blue area). **B** With several immunohistochemical staining applied on contiguous slices for a morphological analysis (hematoxylin–eosin) and for analyzing the expressions of VEGF, Ki-67, integrin αv and integrin β3. **C** Tumor expression of integrin αv and integrin β3 in glioblastoma models

**Fig. 5:**
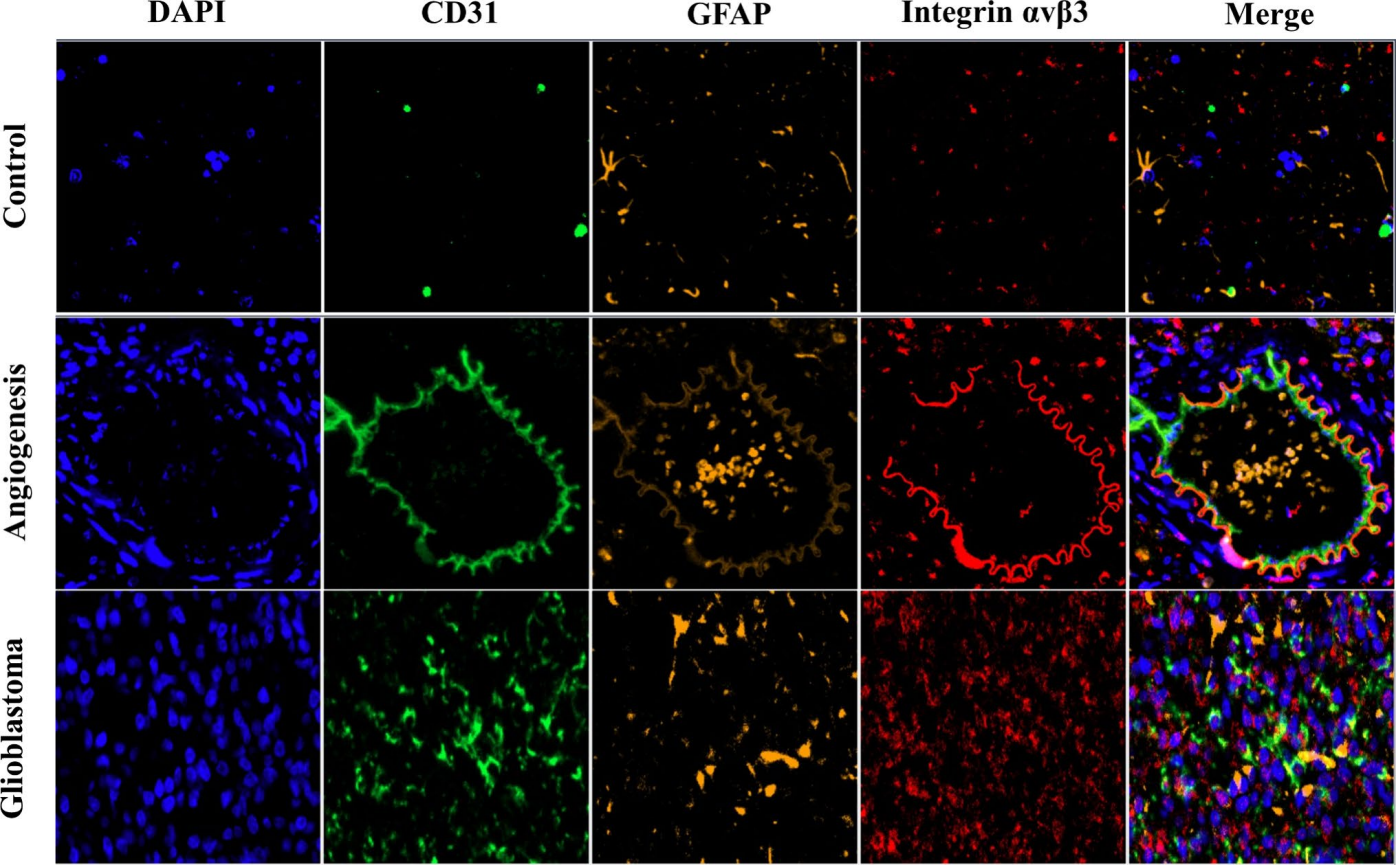
Immunofluorescent microscopy of C6 brain tumor tissue (blue, DAPI/nuclei; red, Integrin αvβ3; green, CD31; orange, GFAP)

Fig. 3 showed that the uptake of [^18^F]Alfatide II by the tumor in the blocking group was significantly reduced. As shown in Fig. 4B, VEGF, Ki-67, integrin αv and integrin β3 expression increased quantitatively in GBM tissues. Western blotting confirmed integrin αv and integrin β3 expression in GBM tissues but not in normal control tissues (Fig. 4C). Furthermore, immunofluorescence staining of tumor tissues in the GBM rat brain confirmed the high expression of integrin αvβ3, tumor angiogenesis marker CD31 and astrocyte activation marker GFAP in glioblastoma region (Fig. 5), with low expression observed in healthy brain tissue. At the same time, the co-expression of the integrin αvβ3, vascular endothelium marker CD31 and GFAP were observed on neovascular endothelial cells in glioblastoma area.

## Discussion

In our study, we focused on the comprehensive comparison of dynamic PET imaging with [^18^F]Alfatide II and [^11^C]MET in orthotopic rat models of GBM. We studied the biodistribution and kinetics of both the radiotracers and validated the findings by using immunofluorescent imaging, western blot, blocking and the immunohistological analysis, which has confirmed αvβ3 integrin expression in GBM. Our results indicate that [^18^F]Alfatide II PET/MR imaging demonstrated superior targeting in glioblastoma, in line with previous findings on variable targeting of primary tumors by [^68^ Ga]RGD (Isal et al. 2018). Notably, we observed a higher tumor-to-normal brain ratio achieved with [^18^F]Alfatide II imaging enhanced the detection and quantification of tumors, compared with that of [^11^C]MET. This suggests a significant potential for [^18^F]Alfatide II in the precise imaging and characterization of glioblastoma, especially in the context of its angiogenic activity. This approach aligns with the broader goal of understanding the complex nature of tumor neovascularization but also potentially enhances the assessment of treatment efficacy and monitoring of disease progression at the microvascular level.

Angiogenesis, the development of new blood vessels, is a critical process in the progression and metastasis of glioblastoma (Verdugo et al. 2022). Extensive research has identified various signaling pathways involved in glioblastoma growth and metastasis, presenting potential targets for therapeutic intervention (Duan et al. 2023). In this context, integrins, particularly involved in cell signaling during angiogenesis, emerge as crucial biomarkers and therapeutic targets. Among these, αvβ3 integrin, predominantly expressed on activated endothelial cells during abnormal tissue growth, plays a significant role in the progression and metastatic spread of glioblastoma (Echavidre et al. 2022; Mezu-Ndubuisi and Maheshwari 2021). This integrin, present on both tumor cells and tumor-associated neovasculature, but more selectively targeted in glioblastoma neovasculature, offers vital insights into the tumor microenvironment (Cheng et al. 2021; Roth et al. 2013). Our study’s use of [^18^F]Alfatide II, a radiotracer targeting αvβ3 integrins in PET imaging, provides noninvasive, real-time evaluation of angiogenesis and its dynamics within the glioblastoma microenvironment (Shao et al. 2020). The radiosynthesis process of [^18^F]Alfatide II is convenient and simple, and can be completed within 30 min, which has great advantages in clinical application (Yu et al. 2015). The results of biological distribution of [^18^F]Alfatide II show that the radioactive accumulation in brain and lung area is relatively low, which also proves the successful application of [^18^F]Alfatide II in patients with lung cancer, breast cancer and brain metastases (Wu et al. 2022; Yu et al. 2015). [^18^F]Alfatide II showed high stability in vivo, and could be quickly removed from blood pool and kidney, and radioactive ligands were slowly metabolized into hydrophilic metabolites (Liu 2015). The time–activity curves (TACs) for the GBM in our study displayed an initial significant increased uptake followed by a phase of plateau. This pattern suggests the absence of trapping or irreversible binding of [^18^F]Alfatide II (Guo et al. 2014a). Reflecting the kinetics of radiotracer uptake in our study, the 2-tissue-compartment model (2TCM) revealed the transport rate of the tracer from blood to tissue, denoted as K_1_, was notably higher in GBM than in the normal brain tissue, suggesting the involvement of processes like increased angiogenesis, heightened tumor permeability, and blood–brain barrier (BBB) disruption (Grkovski et al. 2017). The kinetic parameters K_3_, related to radiotracer binding and cellular internalization or dissociation, was also significantly increased in the tumor (Lindemann et al. 2023), given that ligands like [^18^F]Alfatide II are known to be internalized (Guo et al. 2012).

GBM, characterized by its aggressive nature, has been shown to compromise the BBB’s integrity through a cascade of tumor-induced vascular modification. The blood–brain barrier in GBM exhibits higher permeability compared to healthy brain tissue, attributed to deficient formation, abnormal neovascularization, upregulated transporters, and downregulated tight junction proteins (Liebner et al. 2018; Wu et al. 2021). Furthermore, the C6 glioblastoma model is acclaimed for its fidelity in replicating human GBM pathology, including the pivotal aspect of inducing BBB breakdown (Pournajaf et al. 2024). Research elucidates that the C6 tumor facilitates this breakdown via the secretion of factors like vascular endothelial growth factors (VEGF) and matrix metalloproteinases (MMPs), which collectively undermine the endothelial tight junctions and degrade the basal lamina, thereby compromising the barrier function (Feng et al. 2022; Matsuno et al. 2022). This mechanistic insight into BBB disruption by the C6 model underpins the observed efficacy of [^18^F]Alfatide II in our study, suggesting that its enhanced imaging capability is in part attributable to the increased BBB permeability in GBM. The literature also suggests that [^18^F]Alfatide II possesses the potential to traverse the compromised blood–brain barrier in GBM owing to its specific binding affinity towards integrin αvβ3, which governs migration and invasion of vascular endothelial cells in tumor area, and its cyclic RGD peptides structure that exhibits a heightened attraction for integrins (Liolios et al. 2021). This is particularly relevant for imaging applications, as it implies that [^18^F]Alfatide II could provide enhanced visualization of brain tumors by accumulating in areas where the BBB is disrupted due to tumor growth. Our study’s findings, which indicate a high tumor-to-normal brain ratio for [^18^F]Alfatide II, support this notion.

In our study, ^11^C-MET demonstrated good morphological representation of GBM. However, based on the time–activity curve, the distribution of radioactivity in normal brain tissue was higher for [^11^C]MET than [^18^F]Alfatide II. As a result, its TNR values were significantly lower than those of [^18^F]Alfatide II. This suggests that [^11^C]MET might be less effective than [^18^F]Alfatide II in differentiating between low-grade malignant or benign brain tumor and normal brain tissues. Although the pivotal amino acid imaging agents including [^11^C]MET and [^18^F]Fluoroethyl-L-tyrosine ([^18^F]FET) have shown significant potential in the diagnosis of GBM, their application is still constrained by several important limitations (Lundy et al. 2020; Steidl et al. 2021). Firstly, [^11^C]MET faces significant logistical challenges in clinical applications due to its short half-life (approximately 20 min), limiting its widespread use in medical centers without onsite radioisotope production facilities. Secondly, both [^11^C]MET and [^18^F]FET struggle to differentiate between tumor recurrence and post-radiation changes, as both conditions may show enhanced uptake, increasing the risk of misdiagnosis (Dang et al. 2022). Moreover, [^11^C]MET may also demonstrate increased uptake in non-tumorous lesions, such as inflammation or infection, further complicating its interpretation (Hotta et al. 2019; Mattoli et al. 2021). As for [^18^F]FET, it is slightly less specific as it may accumulate in certain non-malignant brain lesions, such as low-grade gliomas or non-glial pathologies (Fuenfgeld et al. 2020; Maurer et al. 2020). Overall, while [^11^C]MET and [^18^F]FET provide valuable biochemical insights, a more comprehensive assessment and additional diagnostic tools are still required for accurate diagnosis and therapeutic monitoring of GBM.

A notable limitation of our current study is the restricted sample size of the orthotopic Rat Models of GBM utilized, which may not comprehensively represent the diverse heterogeneity characteristic of glioblastomas. This diversity includes a range of biological behaviors and microenvironmental variations. Consequently, the effects observed using [^18^F]Alfatide II in our study might not fully encapsulate the complexity and variability present in a broader spectrum of glioblastoma cases. Further investigations are required to explore these variances in detail, especially regarding the uptake patterns and distribution of [^18^F]Alfatide II in different glioblastoma subtypes. Such studies would be instrumental in understanding the variability in tracer uptake and the expression patterns of target biomarkers like integrin αvβ3 across a more diverse GBM population.

In conclusion, our study presents valuable insights into the application of [^18^F]Alfatide II in the imaging of glioblastoma, demonstrating its potential superiority over [^11^C]MET in terms of tumor-to-normal brain ratio. The significant uptake of [^18^F]Alfatide II demonstrates potential in imaging tumor-associated neovascularization in the context of GBM. Ultimately, this research advances more precise imaging for glioblastoma diagnosis and monitoring, potentially improving the personalized therapies.

### Supplementary Information

Below is the link to the electronic supplementary material.

Supplementary file1 (DOCX 174 KB)

## Data Availability

No datasets were generated or analysed during the current study.
