# Supplementary material for: A comparison study of dynamic [18F]Alfatide II imaging and [11C]MET in orthotopic rat models of glioblastoma
Source: J Cancer Res Clin Oncol. 2024 Apr 22;150(4):208. doi: 10.1007/s00432-024-05688-4 (PMC11035414; doi:10.1007/s00432-024-05688-4)
Supplement: Supplementary file 1 — Supplementary file1 (DOCX 174 KB) [file 432_2024_5688_MOESM1_ESM.docx]

**MATERIALS AND METHODS**

**Biodistribution study:** The biodistribution of [^18^F]Alfatide II and [^11^C]MET in tumor-bearing rats were analyzed. The tumor-bearing rats were tail-intravenously administered approximately 0.74MBq(20μCi) of [^18^F]Alfatide II and sacrificed 1 and 2 hours (n = 3 per time point) after injection(the time point of [^11^C]MET were 15 and 30 minutes). The tumors and major tissue organs (blood, brain, heart, lungs, liver, spleen, blander, kidneys, bowel, muscle, femur and tumor) were collected. Tissues and organs were washed with normal saline and dried, and weighed using an electronic scale. The radioactivity counts of tissues and organs were measured with a γ counter, and the corresponding radioactivity value (%ID/g) was calculated after time attenuation correction.

**Western blot analysis:** Tumor tissue and normal brain tissue were lysed with RIPA buffer containing protease inhibitor cocktail (Servicebio). 4~12% Bis-Tris gradient gel (Invitgen) was prepared with NuPAGE Novex, and an appropriate amount of protein was separated from the total tissue lysate by SDSPAGE, and it was imprinted on the polyvinylidene fluoride membrane (Servicebio). The membrane was sealed with 5% skimmed milk powder, and the different primary antibodies were diluted in the sealing buffer, and incubated at 4℃ overnight. The main antibodies include anti-integrin av and anti-b3(Servicebio). The second antibody is HRP coupled enhanced chemiluminescence (ECL)(Servicebio). Protein bands were detected by ChemiDoc molecular imager (CLINX). The quantitative analysis of Western blot bands was carried out by AIWBwell TM(Servicebio).

**Immunofluorescent staining:** After dewaxing the tumor section, the brain tissue section with antigen repair was treated with QuickBlockTM(cat# P0260, Beyotime, China) blocking buffer. Depending on the antibodies used, PBS was incubated overnight at 4℃ with 1% bovine serum albumin without immunoglobulin or 5% normal donkey serum. The slices were washed with PBS and stained with DAPI for 5min minutes. The main antibodies used are: anti-integrins αvβ3(HUABIO, China), anti-Platelet endothelial cell adhesion molecule-1(CD31)(HUABIO, China) and anti-glial fibrillary acidic protein(GFAP)(HUABIO, China) fluorescence captured on Nikon E100 microscope system. The data were analyzed by Image J software[21].

**RESULTS**

**Biodistribution study:**

As shown in the Supplement Figure 1C, the uptake of [^18^F]Alfatide II by tumor decreased within 120 minutes, from 0.68±0.20 in 60 minutes to 0.41±0.10 in 120 minutes. The uptake of [^11^C]MET by tumor increased within 30 minutes, from 0.81±0.06 in 15 minutes to 0.98±0.06 in 30 minutes(Supplement Figure 1D). Among normal organs, bladder and kidney have the highest uptake of [^18^F]Alfatide II, while liver has the highest uptake of [^11^C]MET. At 120min, the uptake ratio of [^18^F]Alfatide II tumor to brain was 18.62, while at 30min, the uptake ratio of [^11^C]MET tumor to brain was 8.15, and the target to background ratio of [^18^F]Alfatide II was significantly higher than that of [^11^C]MET.


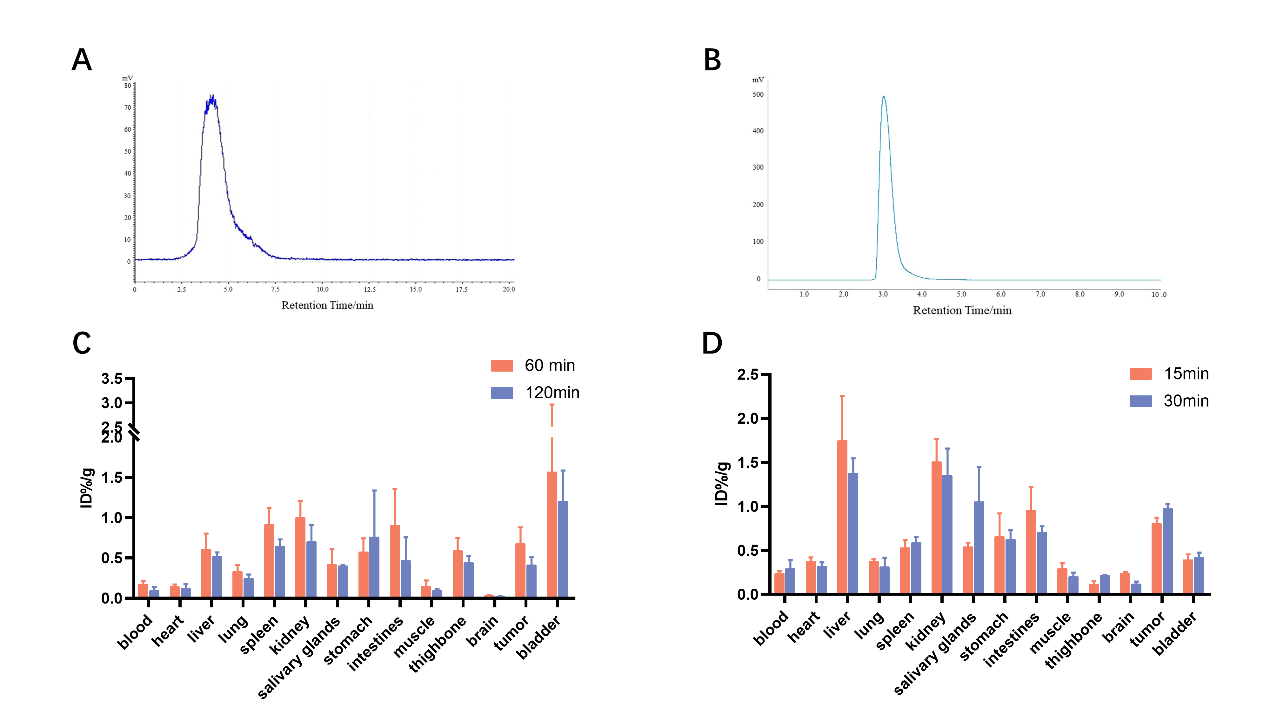


**Supplement Figure.1** (A)HPLC of [^18^F]Alfatide II. (B)HPLC of [^11^C]MET. (C) Uptake values for ex vivo tissues of C6 glioblastoma-bearing rats at 60 and 120 min after administration of 0.74 MBq of [^18^F]Alfatide II (D) Uptake values for ex vivo tissues of C6 glioblastoma-bearing rats at 15 and 30 min after administration of 0.74 MBq of [^11^C]MET (values were expressed as mean ± SD, n=3)
